# CELM: An Ensemble Deep Learning Model for Early Cardiomegaly Diagnosis in Chest Radiography

**DOI:** 10.3390/diagnostics15131602

**Published:** 2025-06-25

**Authors:** Erdem Yanar, Fırat Hardalaç, Kubilay Ayturan

**Affiliations:** 1 Department of Healthcare Systems System Engineering, ASELSAN, 06200 Ankara, Turkey; 2 Department of Electrical and Electronics Engineering, Gazi University, 06570 Ankara, Turkey; firat@gazi.edu.tr (F.H.); kubilay.ayturan@gazi.edu.tr (K.A.)

**Keywords:** cardiomegaly, deep learning, early diagnosis, clinical decision support, medical imaging, automated diagnosis, ensemble learning

## Abstract

**Background/Objectives:** Cardiomegaly—defined as the abnormal enlargement of the heart—is a key radiological indicator of various cardiovascular conditions. Early detection is vital for initiating timely clinical intervention and improving patient outcomes. This study investigates the application of deep learning techniques for the automated diagnosis of cardiomegaly from chest X-ray (CXR) images, utilizing both convolutional neural networks (CNNs) and Vision Transformers (ViTs). **Methods:** We assembled one of the largest and most diverse CXR datasets to date, combining posteroanterior (PA) images from PadChest, NIH CXR, VinDr-CXR, and CheXpert. Multiple pre-trained CNN architectures (VGG16, ResNet50, InceptionV3, DenseNet121, DenseNet201, and AlexNet), as well as Vision Transformer models, were trained and compared. In addition, we introduced a novel stacking-based ensemble model—Combined Ensemble Learning Model (CELM)—that integrates complementary CNN features via a meta-classifier. **Results:** The CELM achieved the highest diagnostic performance, with a test accuracy of 92%, precision of 99%, recall of 89%, F1-score of 0.94, specificity of 92.0%, and AUC of 0.90. These results highlight the model’s high agreement with expert annotations and its potential for reliable clinical use. Notably, Vision Transformers offered competitive performance, suggesting their value as complementary tools alongside CNNs. **Conclusions:** With further validation, the proposed CELM framework may serve as an efficient and scalable decision-support tool for cardiomegaly screening, particularly in resource-limited settings such as intensive care units (ICUs) and emergency departments (EDs), where rapid and accurate diagnosis is imperative.

## 1. Introduction

### 1.1. Clinical Importance of Cardiomegaly and Positioning of AI Systems

**Figure 1:**
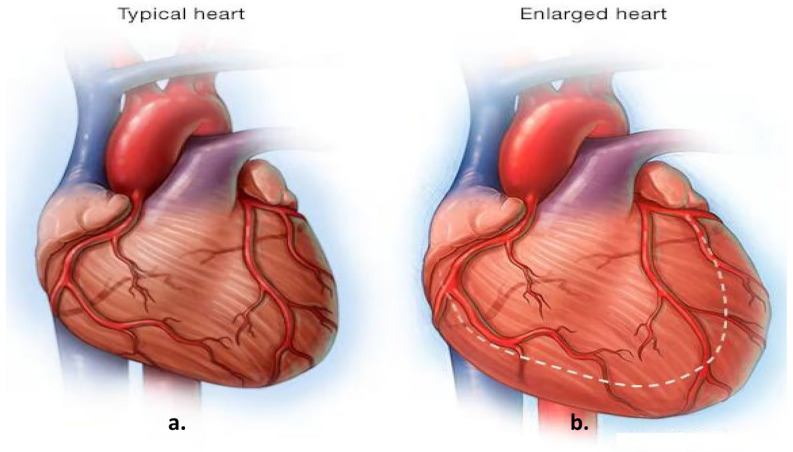
Comparative heart anatomy: (**a**) illustration of a typical heart; (**b**) demonstrating the anatomical changes associated with an enlarged heart [3]. The dotted line indicates the direction of expansion of the heart.

Cardiomegaly, characterized by an enlargement of the heart muscle, is often considered a marker of underlying cardiac conditions and is associated with increased morbidity and mortality. Early diagnosis plays a critical role in improving patient outcomes, particularly given that cardiovascular diseases (CVDs) are among the leading causes of global mortality, accounting for approximately one-third of annual deaths [1]. Early identification and management of cardiomegaly can significantly improve patient outcomes [2]. Imaging techniques hold a central position in the diagnostic process for CVDs, as they facilitate the detection of structural changes in the heart, such as those seen in cardiomegaly. Figure 1, for example, illustrates normal heart anatomy alongside the anatomical changes associated with cardiomegaly [3]. Key clinical indicators of cardiomegaly include dyspnea (shortness of breath), which occurs mainly during exertion or while lying down and is often correlated with elevated biomarkers such as B-type Natriuretic Peptide (BNP) and N-terminal pro B-type Natriuretic Peptide (NT-proBNP), which reflect cardiac strain [4].

Recent advancements in artificial intelligence (AI) and machine learning have opened new opportunities for enhancing diagnostic accuracy in cardiovascular health. Martin-Isla et al. emphasized the importance of cardiac imaging in diagnosing CVDs, highlighting the potential of AI tools to support clinical decision-making [5]. Their study demonstrated how machine learning methods can provide more automated, precise, and early diagnoses. The increasing availability of big data and advanced computational power has further driven rapid progress in AI-powered medical imaging, paving the way for more optimized diagnostic value in existing imaging modalities.

### 1.2. Traditional Diagnostic Methods

The clinical manifestations of cardiomegaly, or an enlarged heart, can vary significantly among individuals. While some patients may remain asymptomatic, others can experience notable symptoms. Cardiomegaly symptoms such as shortness of breath, edema, and arrhythmias are also discussed in detail in Section 1.2. These symptoms, detailed below, highlight this condition’s diverse and complex presentation [6].

**Figure 2:**
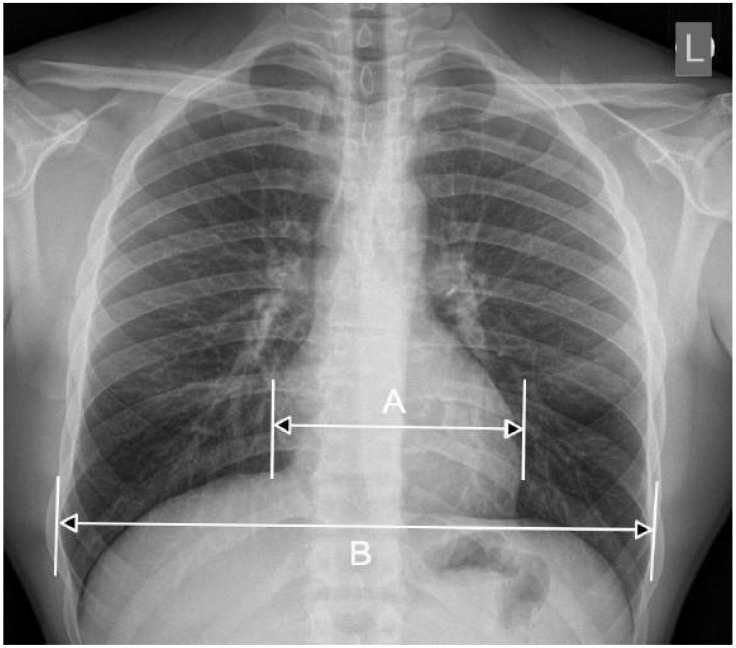
The approach to calculating the CTR involves measuring the relationship between the transverse diameter of the heart (denoted as [A]) and the transverse diameter of the chest cavity (denoted as [B]), as observed on a chest radiograph taken in the posteroanterior (PA) view. The CTR is then computed using the following formula: CTR = A/B [7].

- Physical Examination: Assessment of abnormal heart sounds or dullness upon percussion indicating increased cardiac silhouette.
- ECG: Identifies electrical conduction abnormalities that may suggest chamber enlargement.
- CXR: The visualization of the heart’s size and shape, often used as a preliminary diagnostic tool. Moreover, the Cardiothoracic Ratio (CTR) is a diagnostic metric utilized to identify cardiomegaly (an enlarged heart) in CXRs captured in the posteroanterior (PA) view. In order to determine this ratio, the maximum horizontal diameter of the heart is divided by the maximum horizontal diameter of the thoracic cavity, which is measured at the inner edges of the ribs. An average CTR ranges from 0.42 to 0.50. A CTR exceeding 0.50 is indicative of cardiomegaly, thus signaling the presence of an enlarged heart. For a visual representation, refer to Figure 2, which demonstrates a sample CXR used for CTR calculation.
- Echocardiography: Offers direct measurements of heart chamber sizes and wall motion via ultrasound.

Despite their effectiveness, these methods have limitations regarding availability, cost, and the need for specialized expertise. Details of the challenges are given below:

- Subjectivity: Interpretation of ECGs and CXRs is highly dependent on clinical experience, leading to inter-observer variability.
- Limited Sensitivity and Specificity: Subtle structural changes may be missed, particularly in early-stage cardiomegaly.
- Time and Resource Intensive: Echocardiography requires trained personnel and equipment not always available in emergency or rural settings.
- Diagnostic Delays: Manual assessments can delay decision-making, especially in high-volume clinical environments.

These limitations underscore the need for automated, scalable, and high-precision diagnostic tools. Recent advances in deep learning, particularly convolutional neural networks (CNNs), have significantly improved image-based diagnostics by extracting complex spatial features from chest X-rays. Studies show that AI models can match or even exceed radiologist-level performance in detecting cardiomegaly. By reducing variability and expediting diagnosis, these methods provide reliable clinical decision support, especially in resource-limited settings.

Recent research has demonstrated that machine learning methods and intense learning offer superior accuracy compared to traditional medical imaging diagnostic techniques [8]. Studies conducted by Mohare et al., Que et al., Raghu Kumar et al., and Ayalew et al. have utilized deep learning techniques to diagnose cardiomegaly from X-ray images [9,10,11,12]. Their findings highlight the potential of advanced technology in reliably identifying cardiomegaly. The utilization of sophisticated deep learning algorithms enabled these studies to conduct a comprehensive analysis of X-ray images, thereby identifying patterns linked to cardiomegaly and facilitating the development of an enhanced diagnostic instrument for healthcare professionals.

This study presents a deep learning system using chest X-rays to classify normal and cardiomegaly cases. It combines conventional CNNs, several pre-trained models (VGG16, ResNet50, InceptionV3, DenseNet121/201, AlexNet) with spinal concatenated versions, and Vision Transformers (ViTs) for comparing attention-based approaches in cardiac image classification.

To enhance diagnostic performance and reduce false negatives, the model pipeline includes extensive preprocessing. Specifically, Contrast-Limited Adaptive Histogram Equalization (CLAHE) was applied to improve the visibility of low-intensity anatomical features, while multiplicative speckle noise was suppressed using targeted filtering techniques. These preprocessing steps were critical in enhancing image quality and consistency across datasets.

The goal of this work is to automate the diagnosis of cardiomegaly in a non-invasive, highly accurate, and scalable manner using CXR images. By reducing dependency on manual interpretation and accelerating early detection, the proposed model aims to support clinical decision-making and prevent the progression of undiagnosed cardiomegaly into severe conditions such as heart failure. This study contributes to the literature in the following key ways:

- We propose the CELM (Combined Ensemble Learning Model)—a novel stacking-based ensemble framework that integrates the outputs of VGG16, ResNet50, and InceptionV3 through a shallow meta-classifier to improve cardiomegaly detection from chest X-rays.
- We implement a large-scale evaluation of multiple CNN and Vision Transformer architectures on one of the most comprehensive publicly available CXR datasets (*n* = 50,000) drawn from PadChest, NIH CXR, VinDr-CXR, and CheXpert.
- We report a balanced diagnostic performance with high precision (0.99), specificity (0.92), and AUC (0.90), demonstrating the clinical feasibility of CELM for early cardiomegaly screening.
- We perform a detailed comparative performance analysis with classical and recent models, including VIT-B16 and DenseNet variants, and provide a standardized benchmark in the results section.

We emphasize reproducibility by detailing the architecture, data splits, and performance metrics, and by making the full code and trained models available via GitHub (https://github.com/eyanarai/CELM (accessed on 18 June 2025)) for public reuse and extension.

This section outlines this paper’s structure: Section 2 reviews recent deep learning studies on cardiomegaly; Section 3 details the methodology and dataset; Section 4 covers evaluation methods; Section 5 and Section 6 present results and discussion; Section 7 concludes the study; and Section 8 highlights the study’s contributions.

## 2. Related Works

This section reviews literature on AI advancements in medical imaging for cardiomegaly detection. AI methods, especially CNNs, show strong potential for early, accurate diagnosis from CXRs. Studies have improved sensitivity and specificity via varied architectures and preprocessing, but challenges like dataset variability, model interpretability, and consistency across populations persist. This review highlights key contributions and limitations, framing the innovations of this study.

- Sogancioglu et al. [13] compared anatomical segmentation and image-level classification on frontal CXRs, achieving 97% sensitivity and 90% specificity.
- Yoo et al. [14] applied machine learning to echocardiography, enhancing diagnostic consistency with an accuracy of ~80% and feature visualization support.
- Que et al. [10] introduced CardioXNet using CXR images, reaching 93.75% accuracy and 100% precision.
- Gupte et al. [15] identified cardiomegaly risk factors via clinical studies and developed a model with 94% accuracy and 88% F1-score.
- Candemir et al. [16] fine-tuned pre-trained CNNs for CXR-based cardiomegaly detection, reporting 88% accuracy and 92% recall.
- Raghu Kumar et al. [11] combined 2D and 1D CNNs to classify cardiomegaly with balanced precision, recall, and F1-score of 95%.
- Decoodt et al. [17] proposed a hybrid classical-quantum model achieving 87% accuracy and 93% recall using CQ transfer learning.
- Ayalew et al. [12] used transfer learning (ResNet-50, Inception, DenseNet-169) and visualization techniques (GradCAM), reporting near-perfect metrics (99.8% accuracy).
- Chouhan et al. [18] proposed a stacked ensemble (VGG16, InceptionV3, Xception) for pneumonia detection with >98% accuracy.
- Hussain et al. [19] confirmed that stacking CNNs improves performance over bagging and voting.
- Majkowska et al. [20] benchmarked deep CNN ensembles on radiographs, showing competitive AUCs across multiple thoracic pathologies.

The literature consistently shows that ensemble learning, particularly stacking, outperforms individual models by improving accuracy, robustness, and generalizability. Data augmentation helps mitigate overfitting, especially with limited or imbalanced datasets, while transfer learning significantly boosts diagnostic performance by fine-tuning pretrained models. Building on these insights, this study combines transfer learning and data augmentation within a stacked ensemble of pretrained CNNs for reliable cardiomegaly classification from chest X-rays.

## 3. Materials and Methods

### 3.1. Materials

In this study, we focused on binary classification between cardiomegaly and non-cardiomegaly CXR cases using curated subsets from four large-scale datasets (NIH ChestX-ray14, PadChest, CheXpert, and VinDr-CXR) (Table 1). For the cardiomegaly-positive (target = 1) class, we included all frontal posteroanterior (PA) images labeled with cardiomegaly, regardless of the presence of additional thoracic findings such as pleural effusion or pulmonary edema. For the negative class (target = 0), we included images without a cardiomegaly label, whether labeled as normal or containing other non-cardiac abnormalities (e.g., consolidation, fibrosis, atelectasis). To prevent patient-level data leakage and ensure realistic clinical variation, we included only one PA image per patient, specifically the earliest chronological instance. This approach mimics a real-world setting where patients may present with complex pathologies, and it prevents model bias due to repeated or longitudinal data entries.

#### 3.1.1. CXR NIH Dataset

**Figure 3:**
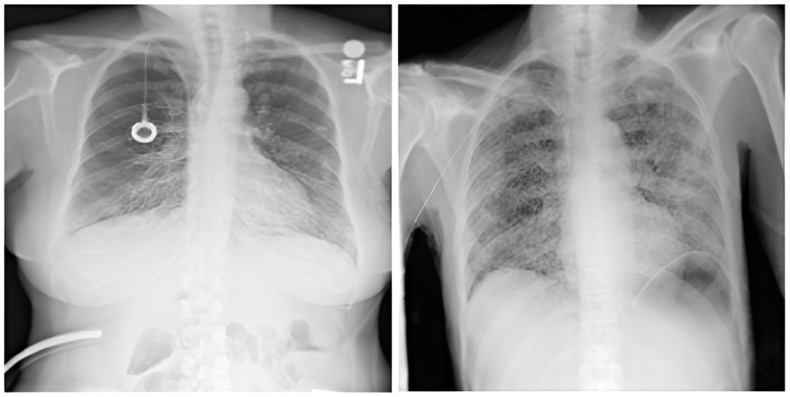
CXR NIH dataset samples [21].

The dataset comprises 112,120 X-ray images with disease labels from 30,805 unique patients [21]. The samples from the CXR NIH dataset are shown in Figure 3. Disease labels were extracted from radiology reports using NLP, achieving over 90% accuracy and supporting weakly supervised learning.

In order to gain a representative insight into the subject matter, the study utilized the CXR NIH dataset. This is a large-scale hospital-grade database comprising 108,948 frontal-view CXR images from 32,717 patients, and it has gained recognition as a valuable resource within the field [22]. The dataset includes images labeled for eight common thoracic diseases using an NLP system applied to radiology reports. While this automated approach enables scalable and consistent labeling with over 90% estimated accuracy, it may occasionally produce ambiguous or incorrect labels.

While the dataset provides a valuable resource for medical imaging research, it is essential to note that the original radiology reports used for labeling are not publicly available. Consequently, direct validation or further refinement of the extracted labels is constrained. Additional details about the labeling process can be found in the freely accessible publication “ChestX-ray8: Hospital-scale CXR Database and Benchmarks on Weakly-Supervised Classification and Localization of Common Thorax Diseases” [23].

This study focused on two subsets of the dataset: images labeled as cardiomegaly and normal. This approach enabled the evaluation of model performance in distinguishing normal from pathological cases. Despite its limitations, the NIH CXR dataset remains a valuable resource for thoracic imaging research.

#### 3.1.2. CheXpert Dataset

**Figure 4:**
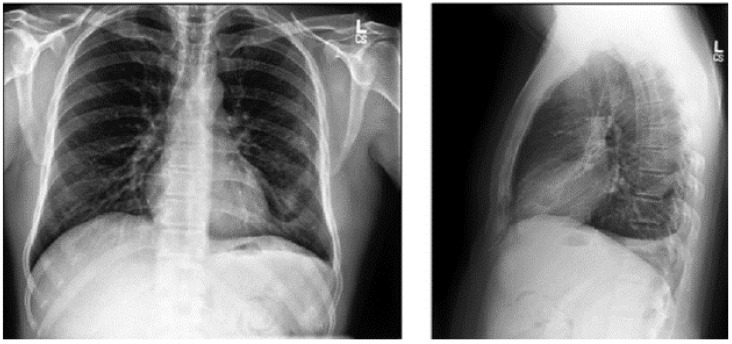
CheXpert dataset samples [24].

This is an extensive and meticulously curated collection comprising 224,316 chest radiographs obtained from 65,240 patients who underwent radiographic examinations at Stanford Health Care facilities over a substantial timeframe spanning from October 2002 to July 2017 [24,25]. The samples from the CheXpert dataset are shown in Figure 4. These examinations were performed across a diverse range of settings, including inpatient and outpatient centers, ensuring the inclusion of a broad spectrum of clinical scenarios and patient demographics. Each radiograph in this dataset has been labeled for 14 distinct, standard chest radiographic observations, aligning with established clinical guidelines and fostering consistency in labeling across the dataset.

#### 3.1.3. CXR VinDr Dataset

**Figure 5:**
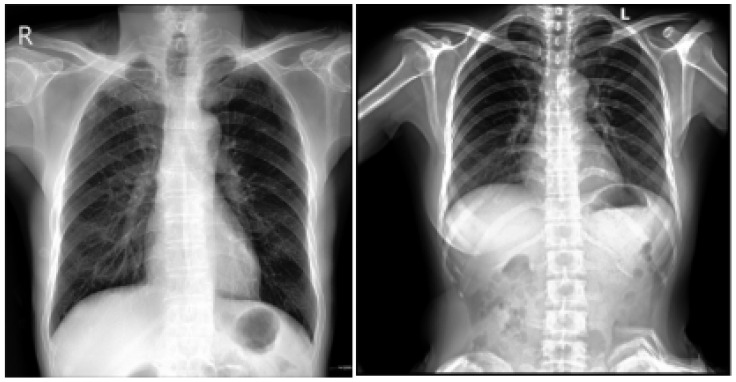
VinDr-CXR Dataset samples [26].

This was developed to provide the research community with a large-scale, high-quality collection of CXR images annotated with detailed and reliable labels [26]. The samples from the CXR VinDr dataset are shown in Figure 5. This dataset was constructed using over 100,000 raw images in DICOM format, which were retrospectively collected from two of Vietnam’s largest and most prominent hospitals: Hospital 108 and Hanoi Medical University Hospital [27]. These facilities contributed to the dataset’s diversity and representativeness by including many cases and patient demographics.

From this initial pool, a carefully curated subset of 18,000 posteroanterior (PA) view CXR scans were selected for publication. These scans are of particular value as they facilitate the visualization of critical radiological results and the categorization of common thoracic diseases. This provides researchers with a robust dataset that can be used for several applications, including disease detection and model validation.

A team of 17 highly experienced radiologists, each with at least eight years of professional expertise, meticulously annotated the images. Their annotations cover 22 critical findings (local labels) and six diagnostic categories (global labels). The local labels correspond to specific findings localized on the images with bounding boxes, enabling precise identification of pathological regions. The global labels, on the other hand, align with the “Impressions” section of a standard radiology report, summarizing the overall diagnostic conclusions. These local and global labels provide a comprehensive structure that mirrors the organization and detail typically found in professional radiology reports. This ensures the dataset’s usability for various research purposes in medical imaging and beyond.

#### 3.1.4. PadChest Dataset

**Figure 6:**
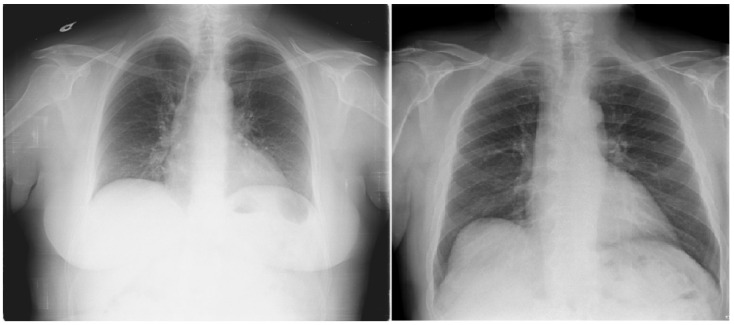
PadChest dataset samples [28].

This is a comprehensive and valuable resource for medical imaging research, consisting of over 160,000 CXR images obtained from 67,000 patients [28,29]. The samples from the PadChest dataset are shown in Figure 6. These images were collected between 2009 and 2017 at San Juan Hospital in Spain, representing various cases and clinical scenarios [30]. The dataset includes images captured in six position views, providing versatility for various radiological studies. Additionally, it contains extensive metadata detailing information about image acquisition techniques and patient demographic characteristics, further enhancing its utility for diverse research applications.

Each image in the dataset is accompanied by radiology reports interpreted and documented by experienced radiologists at San Juan Hospital. These reports have been annotated with 174 distinct radiographic findings, 19 differential diagnoses, and 104 anatomical locations. The findings are organized within a hierarchical taxonomy and systematically mapped to standardized medical terminologies, specifically the Unified Medical Language System (UMLS) [29]. This mapping ensures consistency and facilitates interoperability with other medical datasets and systems, making the dataset an invaluable tool for advancing machine learning applications in healthcare.

The annotation process involved a rigorous combination of manual and automated efforts to ensure both accuracy and scalability. Overall, 27% of the reports were manually annotated by trained physicians, who provided meticulous and high-quality labels. An automated labeling approach was employed for the remaining reports, utilizing a supervised method based on a recurrent neural network equipped with attention mechanisms. This hybrid labeling strategy balances precision and efficiency, resulting in a robust dataset that is well-suited for training and validating machine learning models to improve diagnostic accuracy and decision support in medical imaging.

To ensure robust training and avoid model bias toward the majority class, we implemented a class balancing strategy across the datasets. Since the original datasets contained uneven distributions of cardiomegaly-positive and normal chest X-ray (CXR) images, a class-wise downsampling approach was applied.

The following was performed for each dataset:

- An equal number of normal and cardiomegaly-positive images were selected.
- If the number of cardiomegaly cases was lower than that of the normal cases (as in CXR NIH and VinDr-CXR), an equal-sized subset of normal images was randomly selected.
- Conversely, in datasets with more cardiomegaly images (e.g., CheXpert), the majority class was downsampled to match the minority class to maintain a 1:1 class ratio.

This strategy ensured the following:

- A balanced class distribution, reducing the risk of biased learning.
- An improved generalization and stability during training.
- A fair evaluation of model performance across both classes.

To further improve robustness, the selected samples were shuffled and split into training, validation, and test sets in a stratified manner, preserving the class balance in each subset as shown in Table 2, combining data from four distinct sources: the PadChest, CXR NIH, CXR VinDr, and CheXpert datasets.

### 3.2. Methods

To build a robust and generalizable model for cardiomegaly detection, this study incorporated data augmentation and transfer learning as foundational strategies. This techniques were employed to enhance model performance, reduce overfitting, and address limitations related to dataset size and variability [29].

A total of 54,836 chest X-ray (CXR) images were initially collected from the PadChest, CXR NIH, VinDr-CXR, and CheXpert datasets. After rigorous quality filtering and class-balancing, a curated dataset of 50,000 images was finalized, containing equal numbers of cardiomegaly-positive and normal cases [29].

Quality filtering excluded low-resolution, poorly exposed, or truncated images. To enforce a 1:1 class ratio, randomly selected samples from the overrepresented class were removed. The resulting dataset was split into 40,000 training, 5000 validation, and 5000 test images as shown in Table 3.

In particular, the deliberate exclusion of images with poor resolution, noise, or improper exposure may result in an overly optimistic performance profile. In clinical settings, such imperfections are common; thus, the current model’s generalizability to routine hospital data must be tested in follow-up studies.

While this curated dataset supports more consistent model training, we acknowledge that it does not fully reflect the variability found in real-world clinical practice. Excluding technically suboptimal images may reduce generalizability, as actual hospital workflows often involve images with diverse exposure, noise, and positioning artifacts. Furthermore, although non-cardiomegaly cases included other thoracic pathologies (e.g., consolidation, fibrosis, atelectasis) and cardiomegaly cases included findings such as pleural effusion and pulmonary edema, both groups were still partially curated. As such, the dataset does not encompass the full spectrum of clinical heterogeneity. Future research will focus on expanding data diversity to include a wider range of imaging quality and comorbidities, enabling better representation of real-world diagnostic scenarios.

Transfer learning was utilized by leveraging pre-trained CNNs such as VGG16, ResNet50, InceptionV3, DenseNet121, DenseNet201, and AlexNet. These models, originally trained on large-scale datasets like ImageNet, offer deep hierarchical representations that can be fine-tuned for medical imaging tasks. By adapting these architectures to the chest radiography domain, we significantly accelerated training and improved classification accuracy, especially given the complexity and subtlety of cardiomegaly features on chest X-rays (CXRs).

In parallel, data augmentation techniques were applied during training to artificially expand the dataset and introduce greater variability. Augmentations included random rotation, zoom, and horizontal flipping, simulating real-world variations in imaging conditions. Additionally, CLAHE was applied to enhance local contrast, improving feature visibility in low-intensity regions—common in medical images. These preprocessing steps collectively aimed to enhance model generalization and reduce bias toward specific imaging conditions.

While combining datasets enables the model to learn from a broad distribution, the train-test split was performed after merging the datasets. As such, domain-specific effects between data sources may not be fully captured. Future work will explore cross-dataset validation to better simulate real-world deployment scenarios involving domain shift.

#### 3.2.1. Preprocessing

Prior to model training, all chest X-ray (CXR) images were resized to a resolution of 224 × 224 pixels, which ensures compatibility with standard CNN architectures while balancing computational efficiency [29]. Pixel values were normalized to a [0, 1] range to facilitate faster convergence during training. To reduce noise—particularly speckle and multiplicative noise commonly present in radiographic data—image smoothing filters were applied during preprocessing. This ensured that finer anatomical features relevant to cardiomegaly detection, such as heart borders and lung field clarity, remained intact while minimizing distortions that could hinder model performance [29].

#### 3.2.2. Clinically Aware Data Augmentation

Data augmentation was carefully designed to increase dataset variability while preserving the thoracic anatomical structures critical for cardiomegaly assessment.

Horizontal flipping was applied with a 50% probability, as frontal chest radiographs maintain diagnostic validity regardless of left–right symmetry in most cardiomegaly cases [29].

Rotation was constrained to a small range of ±5 degrees, as larger rotations could deform key structures such as the heart silhouette or mediastinum, which are essential for accurate diagnosis.

Zoom augmentation was applied within a narrow range of 95% to 105%**,** simulating realistic changes in radiographic magnification during image acquisition while avoiding the disproportionate enlargement of the heart or lungs.

Translation was limited to ≤5% of the image width or height (i.e., maximum shifts of approximately ±10 to 12 pixels) along the x- and y-axes to simulate minor variations in patient positioning without displacing core anatomical features.

Additionally, combinations of flipping and translation were used selectively to further enhance spatial diversity without compromising clinical interpretability [29].

#### 3.2.3. Contrast Enhancement with CLAHE

To improve the visibility of thoracic anatomical landmarks in low-contrast images, Contrast-Limited Adaptive Histogram Equalization (CLAHE) was applied. The enhancement process was conducted on the luminance channel after converting the image from RGB to the LAB color space. CLAHE was implemented using a clip limit of 2.0 and a tile grid size of 8 × 8, parameters optimized to enhance local contrast while preventing the over-amplification of noise. This method improved contrast along subtle cardiac contours and lung borders, thereby aiding the model in detecting cardiomegaly-related features. Furthermore, to ensure consistent preprocessing across all datasets, a binary K-Means clustering algorithm was used to detect white-background images, which were then inverted to match the standard dark-background format used in CXR interpretation [29].

**Figure 7:**
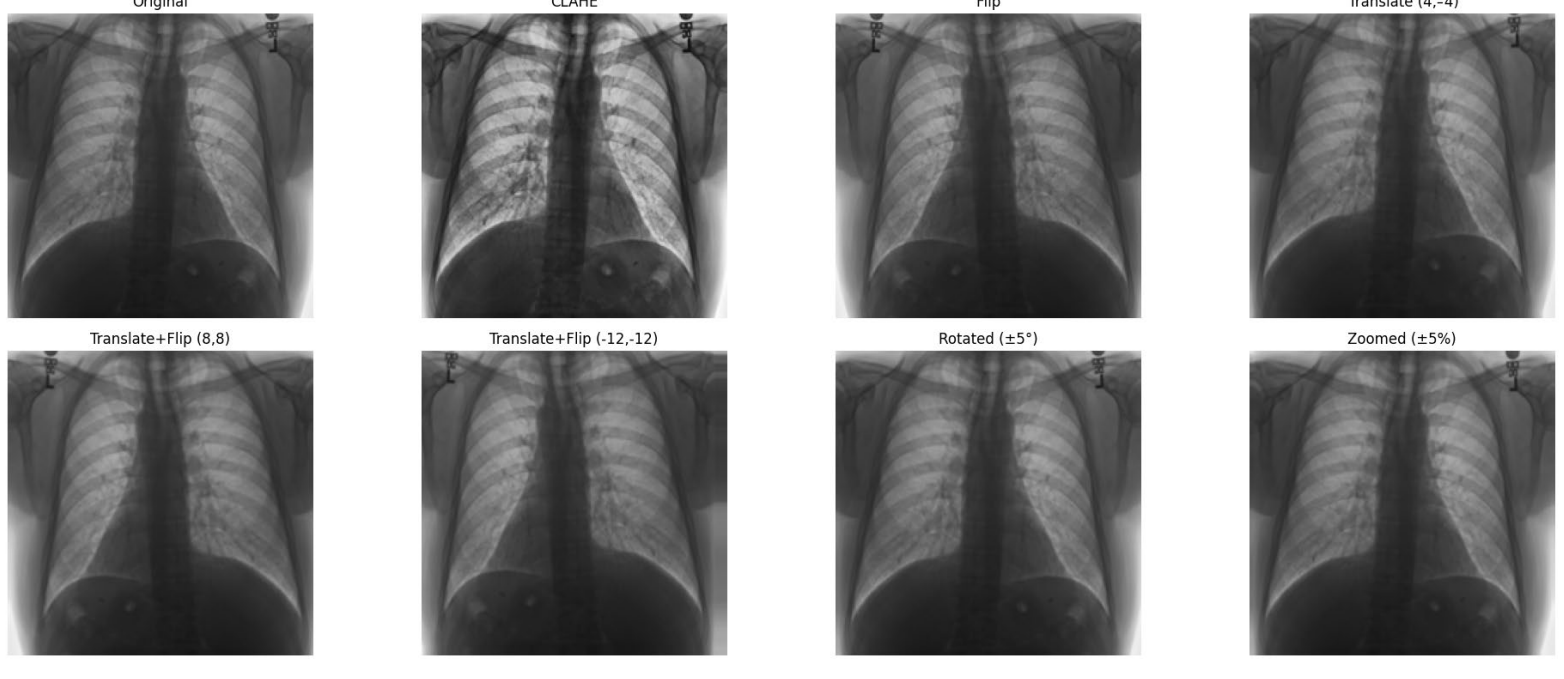
Data augmentation example.

Figure 7 shows examples of images to which data augmentation techniques have been applied. Table 4 provides a detailed explanation of the types of data augmentation, their settings, and their clinical purposes.

As a result of data augmentation, the dataset was expanded to comprise a total of 50,000 images, evenly distributed between cardiomegaly cases and other categories (e.g., no findings and various other diseases) sourced from four primary datasets. Of these, 40,000 images were designated for training, 5000 for validation, and 5000 for testing. A detailed breakdown of the input images is provided in Table 5.

### 3.3. Model Architectures

To promote reproducibility and technical clarity, we provide comprehensive architectural details for all models evaluated in this study. This includes clear definitions of the CNN and Vision Transformer backbones and the proposed stack-based ensemble (CELM) structure, all of which are presented in detail. We explored several CNN and Vision Transformer architectures to determine the most effective model for diagnosing cardiomegaly from CXRs [29]. The models included were as follows:

- Vision Transformer (ViT) is a deep learning model for image recognition introduced by Google Research in 2020 as a CNN alternative. Instead of convolutions, it uses self-attention (Transformer) to analyze images. ViT-B/16 refers to the Base model that splits images into 16 × 16 patches and processes them with a Transformer. The architecture consists of three main parts:
- Patch Embedding: An input image (e.g., 224 × 224 pixels) is divided into non-overlapping patches of size 16 × 16. Each patch is flattened into a vector and embedded into a feature space. The embedding also includes position encodings to retain spatial information.
- Transformer Encoder: These patch embedding is fed into a standard Transformer encoder (similar to NLP models like BERT). Self-attention layers help the model understand long-range dependencies between patches.
- Classification Head: A class token is added to represent the entire image. The final output is processed by an MLP (multi-layer perceptron) to generate classification scores.

**Figure 8:**
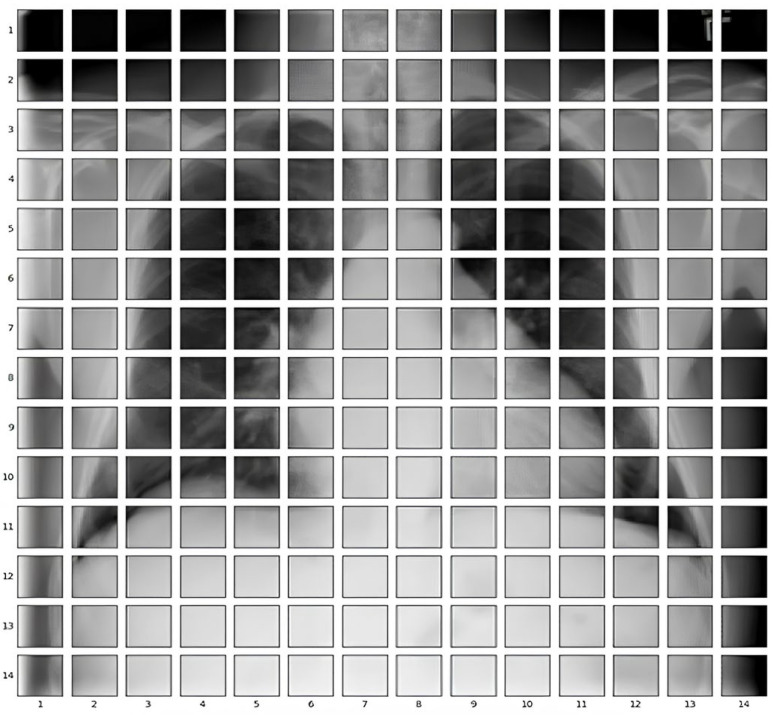
ViT Example Image.

**Figure 9:**
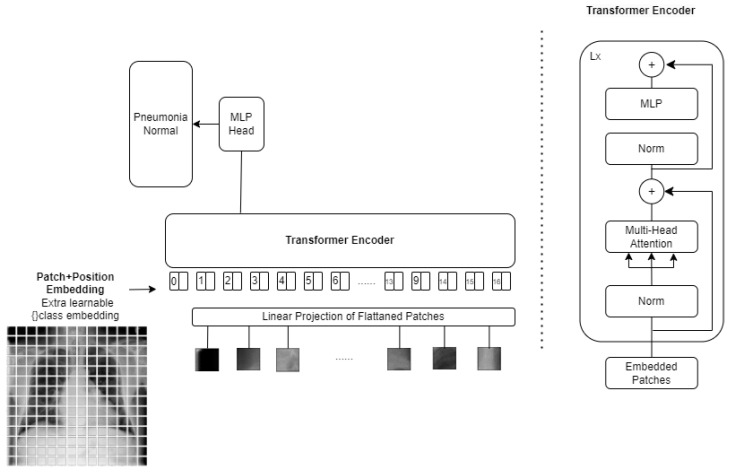
ViT architecture.

An example image with ViT applied is shown in Figure 8. Table 6 also shows the patch configuration for image processing. The total model consists of 12 transformer encoder layers, 768-dimensional hidden representation, 12 self-attention heads, and ~86 million total parameters, as shown in Figure 9.

**Figure 10:**
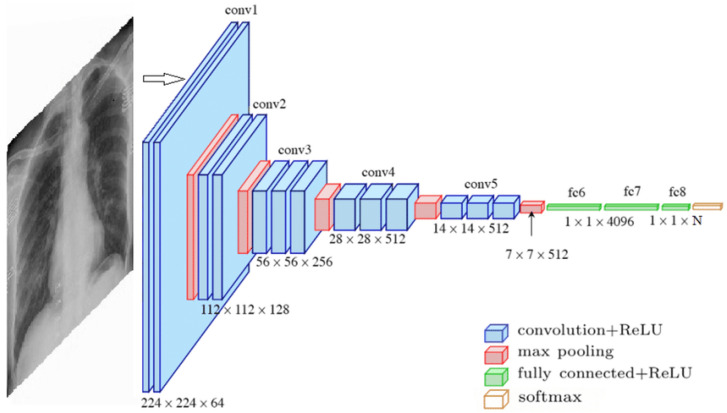
VGG-16 architecture [31].

- VGG16 is a deep CNN known for its simplicity and depth, making it a strong baseline for image classification tasks. It is a 16-layer CNN with 13 convolutional layers and 3 fully connected layers, designed to process input images of 224 × 224 pixels. The first two fully connected layers contain 4096 channels each, while the third includes 1000 channels. Training is performed over 16 epochs, with 326 steps per epoch, to optimize the accuracy and processing efficiency. The architecture of the VGG16 model is shown in Figure 10.

- ResNet50: The Residual Network 50-layer (ResNet50) architecture is a powerful model for image classification, capable of achieving state-of-the-art results when trained on large datasets. Its key innovation lies in using residual connections, which enable the network to learn residual functions that map inputs to outputs effectively. These connections allow the ResNet50 architecture to build significantly deeper architectures without encountering vanishing gradient issues, a challenge in traditional deep networks. The ResNet50 architecture is composed of four main components:
  1. Convolutional Layers.
  2. Identity Blocks.
  3. Convolutional Blocks.
  4. Fully Connected Layers.

Each component is vital in processing the input image and performing accurate classification. A detailed breakdown of the ResNet50 architecture is shown in Table 7.

**Figure 11:**
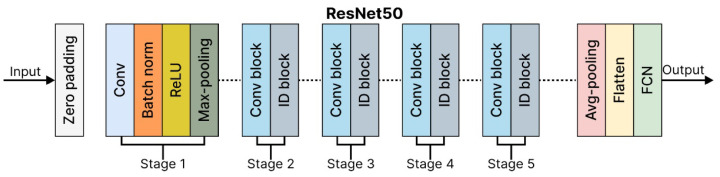
ResNet50 architecture [31].

ResNet50 is highly efficient for deep learning tasks, balancing accuracy and computational complexity, and is widely used in research and industry. ResNet50Architecture is shown in Figure 11.

**Figure 12:**
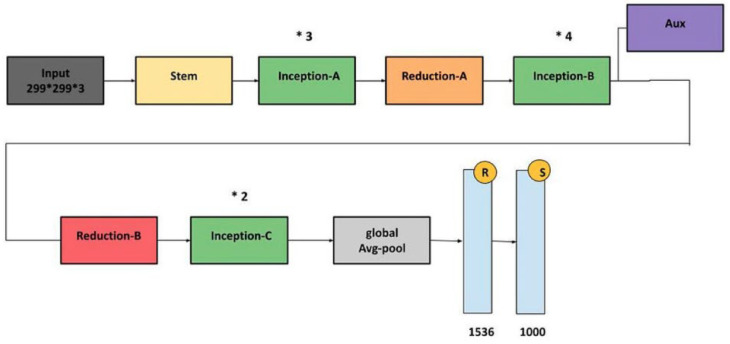
InceptionV3 architecture [32].

**Figure 13:**
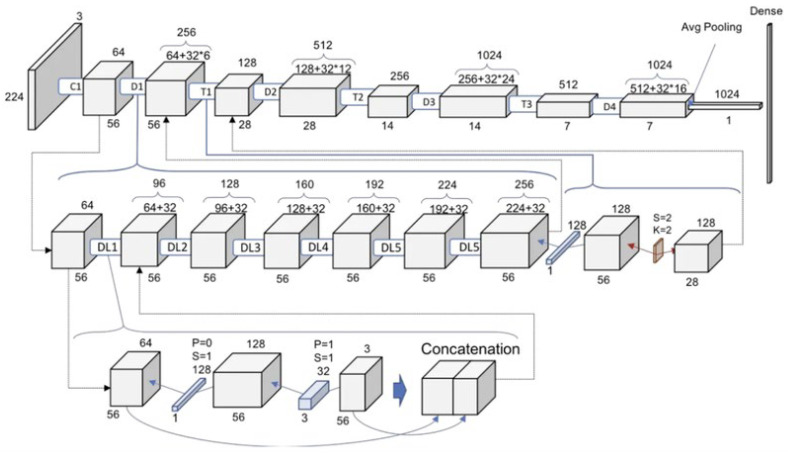
DenseNet121 architecture [33].

- InceptionV3: InceptionV3 is an efficient and widely adopted convolutional neural network architecture designed for image classification tasks. It incorporates several key innovations to enhance accuracy while reducing computational complexity. Among these are label smoothing, which mitigates overfitting by preventing overconfidence in predictions, and convolutional factorization, where large 7 × 7 filters are replaced with more efficient combinations of 1 × 7 and 7 × 1 convolutions. The model also includes an auxiliary classifier during training, which provides intermediate supervision to combat the vanishing gradient problem and improve convergence, though it is removed during inference. InceptionV3 applies batch normalization and gradual grid size reduction to stabilize training and preserve spatial information. Its modular structure consists of inception modules that extract multi-scale features through parallel convolutions with various filter sizes (1 × 1, 3 × 3, and 5 × 5), enabling rich and diverse feature representations. Overall, InceptionV3 offers a strong balance between computational efficiency and classification performance. The architecture is illustrated in Figure 12.

- DenseNet121: This constitutes a standard CNN, whereby the input image is passed sequentially through the network layer by layer, thus enabling the production of an output predicted label. This process is illustrated in Figure 13.

Each convolutional layer, except the first, receives the output feature map of the previous layer as its input and produces its feature map, which is then passed to the next layer. A network with L layers has L direct connections, each linking a layer to its immediate successor. In contrast, the DenseNet architecture introduces a novel approach by connecting each layer to every other layer within the network, giving rise to the term “Densely Connected Convolutional Network.” A DenseNet with L layers has L(L + 1)/2 direct connections. Each layer takes the feature maps from all preceding layers as input and, in turn, contributes its feature maps as input to all subsequent layers. This structure significantly enhances the flow of information and gradients throughout the network.

**Figure 14:**
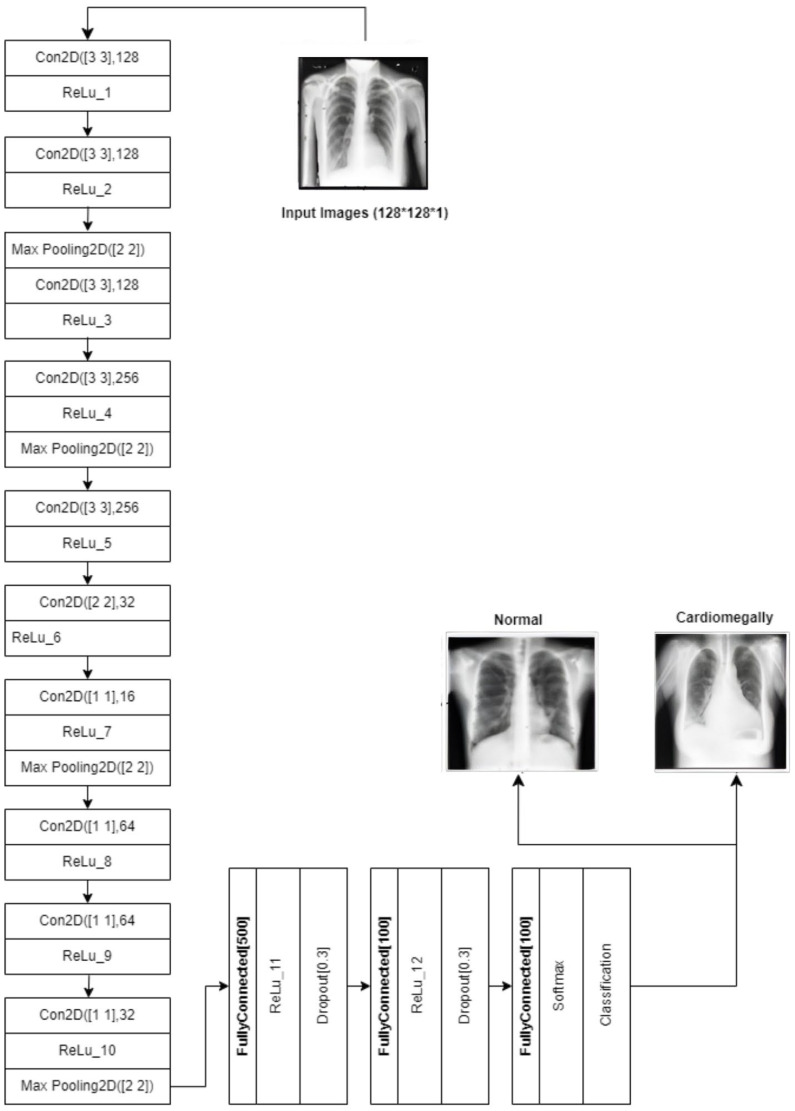
Diagram of the customized CNN.

- CNN: The CNN architecture is designed to detect cardiomegaly in CXR images by progressively extracting features using 3 × 3 convolutional layers (128 and 256 filters). It then uses 1 × 1 point-wise convolutions and a 2 × 2 convolutional block to reduce spatial dimensions while preserving key patterns. The resulting feature maps are flattened and passed through fully connected layers with 500 and 100 neurons, with 30% dropout applied between layers to prevent overfitting. The final layer outputs classification probabilities using sigmoid or softmax activation. This design efficiently combines feature extraction, dimensionality reduction, and regularization. The architecture is shown in Figure 14.

**Figure 15:**
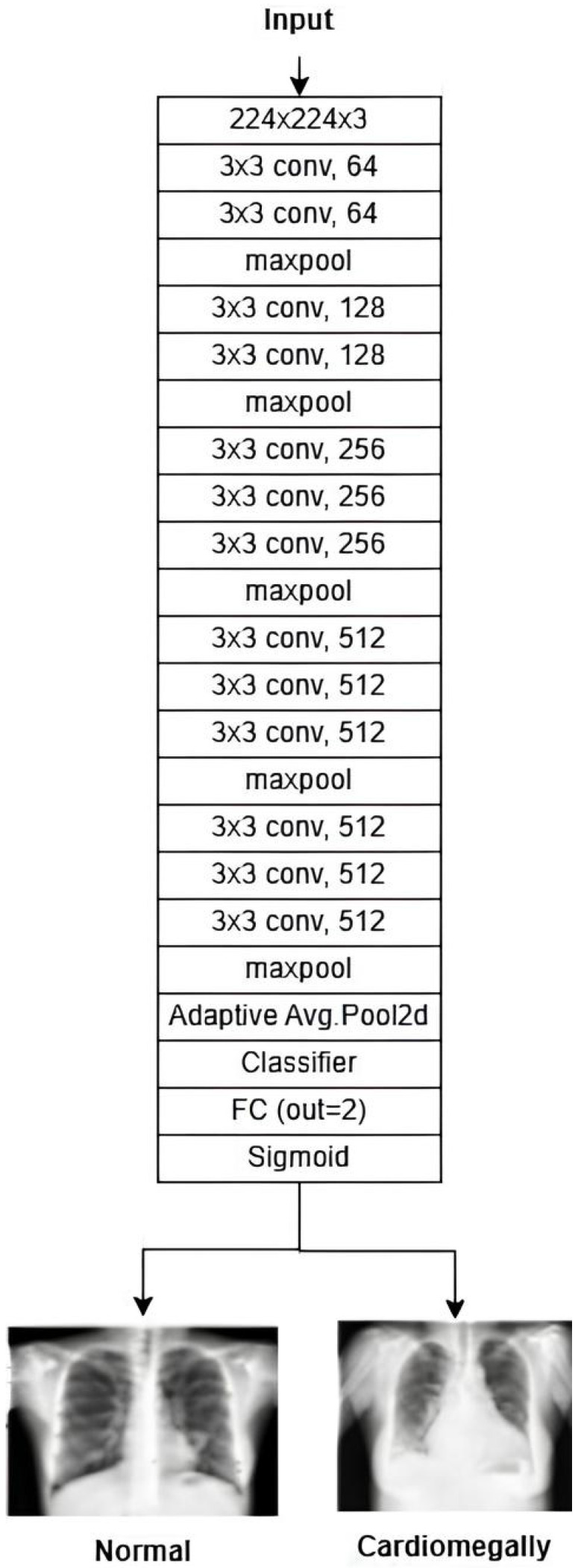
The structure of the adapted VGG16-based model.

**Figure 16:**
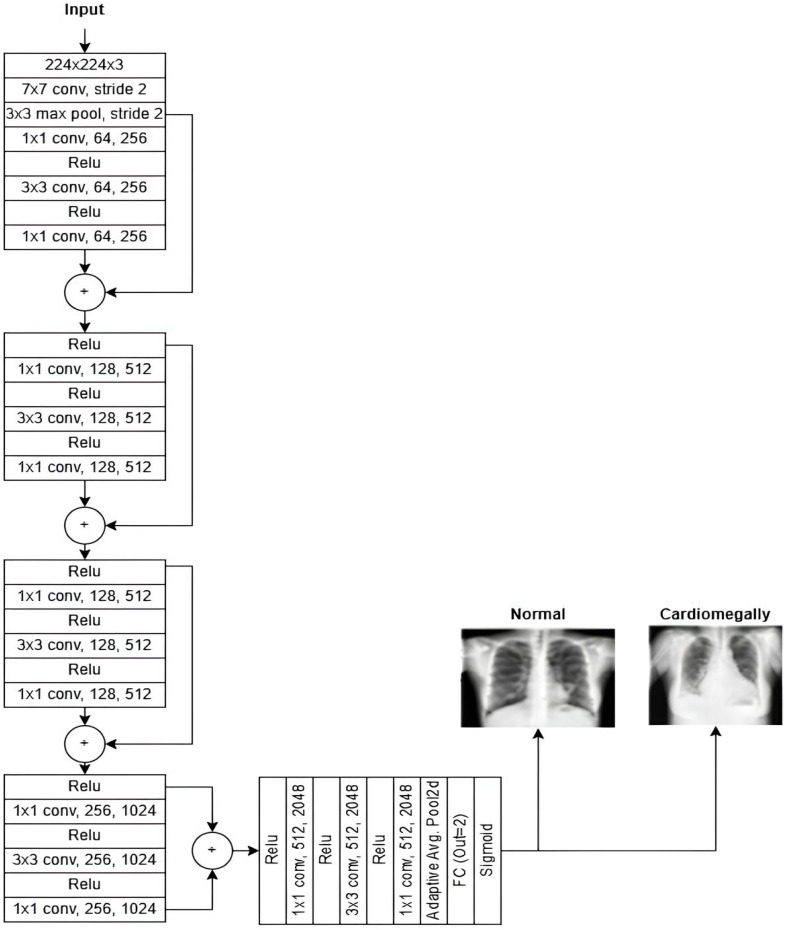
The structure of the adapted ResNet50-based model.

**Figure 17:**
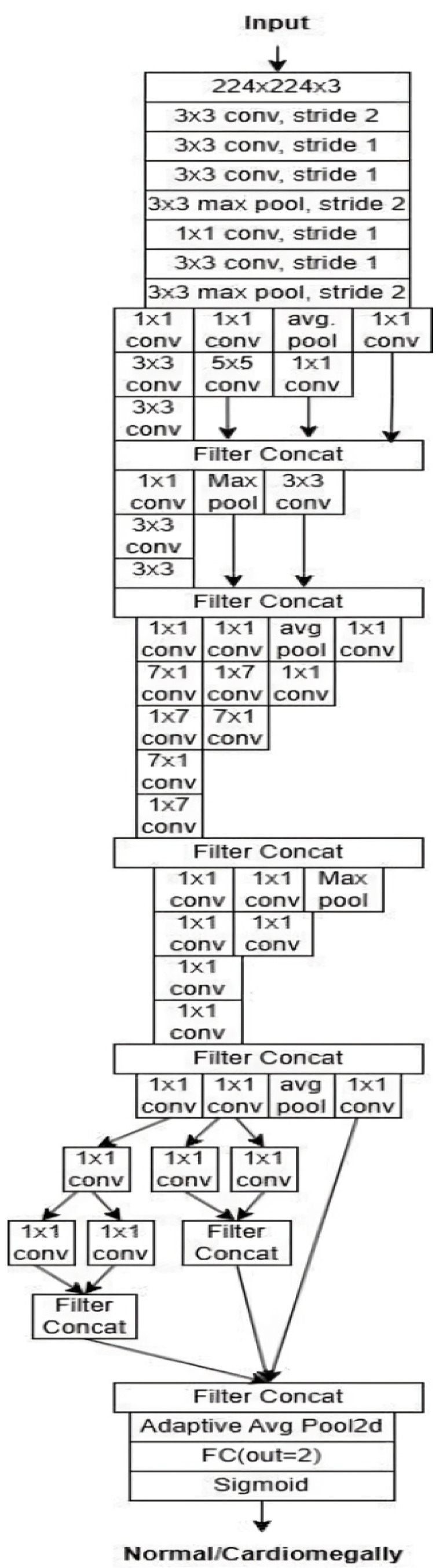
The structure of the adapted InceptionV3-based model.

The subsequent step is to diagnose the underlying condition, following the creation of a CXR mask encompassing the affected area and a diagnosis of cardiomegaly. This is accomplished through the training of disparate data samples and the development of a model that is capable of diagnosing test data. Figure 15 (VGG16), Figure 16 (ResNet50), and Figure 17 (InceptionV3) give customized versions of well-known classification models.

**Figure 18:**
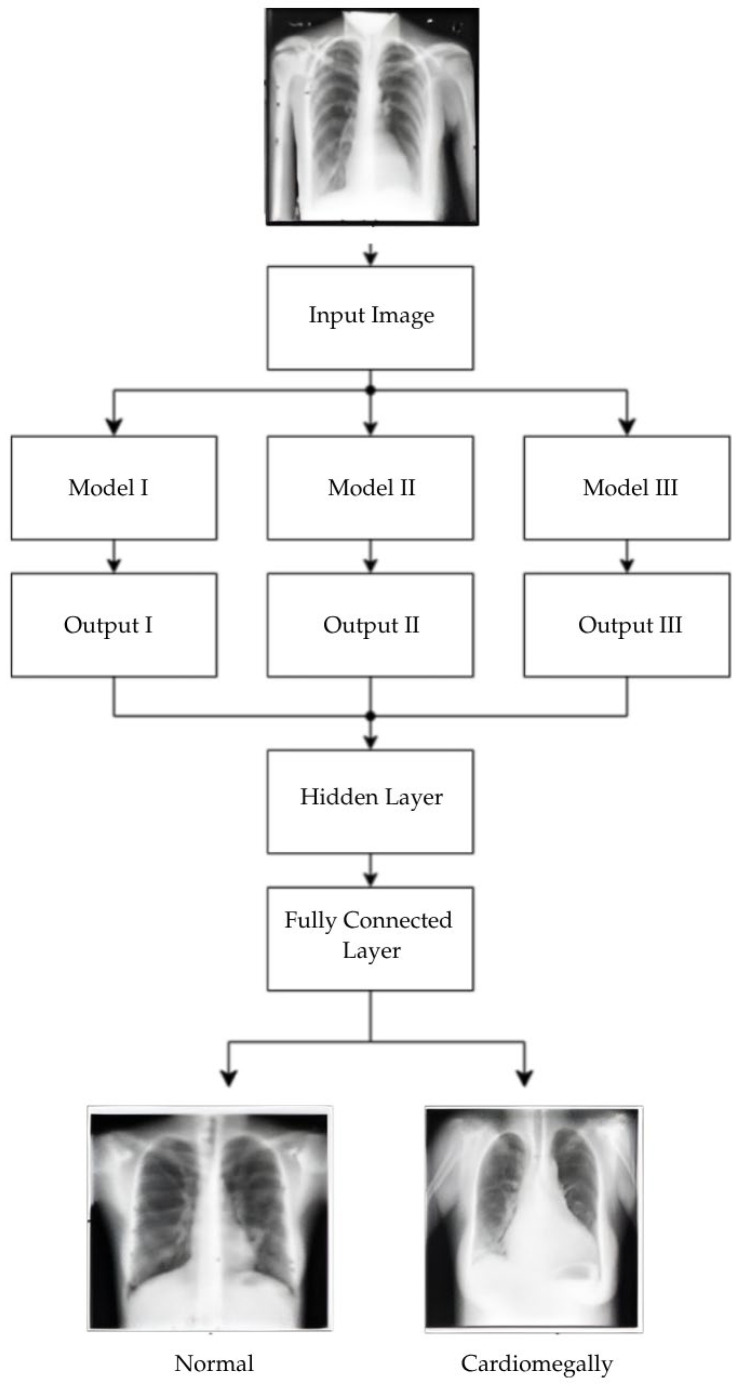
Workflow of our ensemble learning model.

**Figure 19:**
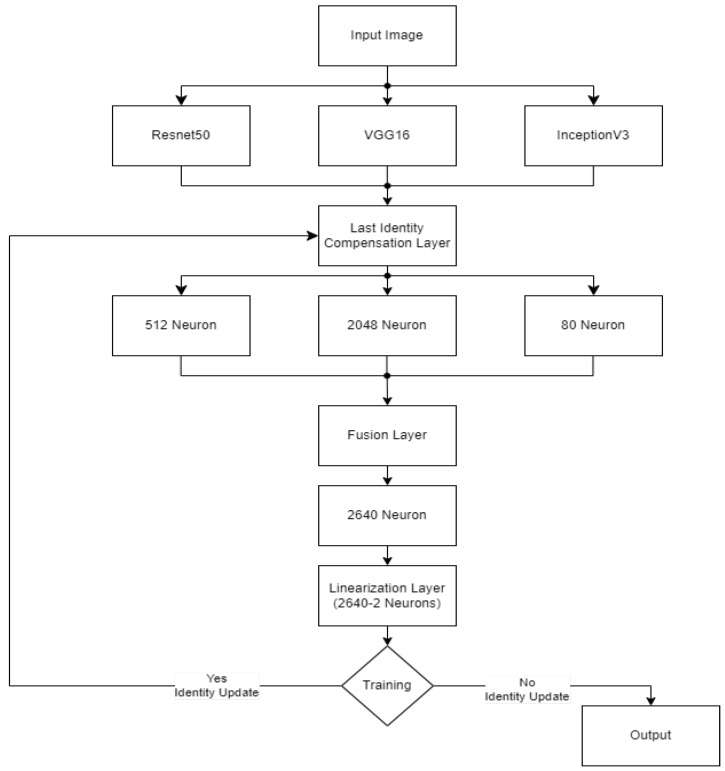
Details of our ensemble learning model.

This study uses stacking, an ensemble learning model. Figure 18 and Figure 19 shows the workflow of the ensemble learning model used in this study. Stacking represents a sophisticated ensemble learning technique that combines predictions from multiple machine learning models to improve classification. In contrast to other ensemble methods, such as bagging or boosting, stacking is designed to integrate the strengths of diverse base models by training a secondary model, frequently referred to as the meta-learner, on the outputs of these base models. This approach enables the capture of more complex patterns and relationships within the dataset. In healthcare, stacking has been demonstrated to be a particularly valuable technique, particularly in diagnosing diseases where the data is heterogeneous and the features are diverse.

To illustrate, a recent study employed a multi-modal stacking ensemble approach to diagnose cardiovascular diseases using ECG data. This approach integrated predictions from deep learning models trained on scalograms and grayscale ECG images. Using a logistic regression model as the meta-learner to combine the base predictions resulted in a notable improvement in performance metrics, with an area under the curve (AUC) of 0.995, compared to the results obtained from individual models or more straightforward ensemble techniques. The stacking approach proved particularly efficacious in leveraging the complementary strengths of different data modalities to enhance diagnostic accuracy [34,35].

Another study used stacking to combine multiple classifiers to predict coronary heart disease. This was particularly effective in handling echocardiographic data’s noisy and heterogeneous nature. The study demonstrated that stacking outperformed other methods by capturing hidden interactions among clinical and imaging features, thereby showcasing its ability to provide more precise and reliable disease diagnosis.

These examples highlight the potential of stacking to address challenges in healthcare, such as integrating diverse data sources and improving prediction accuracy, thus making it a promising tool for advancing medical diagnostics [36].

The CELM (Cardiomegaly Ensemble Model) utilizes a stacking-based architecture that integrates three independently trained deep learning models—ResNet50, VGG16, and InceptionV3—to enhance the accuracy and robustness of cardiomegaly diagnosis from posteroanterior (PA) chest X-ray images. As illustrated in Figure 18, each model is coupled with a distinct fully connected (FC) mechanism: Spinal Fully Connected (Spinal FC) layers for ResNet50 and InceptionV3, and a standard FC layer for VGG16.

The analysis of the CELM, designed for classifying Chest PA (posteroanterior) X-ray images as normal or abnormal (cardiomegaly), reveals that three distinct CNN-based models are utilized. In the corresponding figure, “data” refers to the complete dataset of shoulder bone X-ray images. “Model I” represents the ResNet50 model integrated with SpinalNet FC, “Model II” corresponds to the VGG16 model with a Standard FC layer, and “Model III” denotes the InceptionV3 model combined with SpinalNet FC. The outputs of these models, labeled as “Outputs 1–3,” contribute to the final “Prediction,” which categorizes the images as either normal or abnormal (cardiomegaly).

Independent feature extraction and prediction part, each base model accepts a 224 × 224 × 3 input image and extracts deep visual features through its convolutional and pooling layers [29]. Rather than directly outputting classification labels, the models are modified to generate single scalar probability values using sigmoid activation functions, indicating the likelihood of the input image being classified as cardiomegaly.

Let the following be defined:

- P_1_: the output from VGG16.
- P_2_: the output from ResNet50.
- P_3_: the output from InceptionV3.

These values are soft predictions and are not thresholded at this stage.

In the feature concatenation part, the scalar outputs from the three models are concatenated into a 1 × 3 feature vector:

(1) $$P=[P_{1},P_{2},P_{3}]\in R^{1\times 3}$$

This unified prediction vector becomes the input to the meta-classifier, which learns how to optimally combine the base predictions to improve decision-making performance.

Meta-Classifier Architecture:

As depicted in Figure 19, the meta-classifier is a fully connected feed-forward neural network that transforms the 1 × 3 input vector into a final binary decision. The architecture includes the following layers:

- Input layer: Accepts the concatenated vector [P_1_, P_2_, P_3_].
- Dense hidden layer: Contains 8 neurons, activated by the ReLU function.
- Dropout layer: Applied during training to reduce overfitting and promote generalization.
- Output layer: A single neuron with sigmoid activation that outputs the final probability of cardiomegaly.

The entire meta-classifier is trained using binary cross-entropy loss and optimized with the Adam optimizer.

In final prediction and thresholding stage, the sigmoid output from the meta-classifier is computed as follows:

(2) $$\mathrm{Pfinal}=\sigma (w_{1}P_{1}+w_{2}P_{2}+w_{3}P_{3}+b)$$

where

- w_1_, w_2_, and w_3_ are the learned weights;
- b is the bias term;
- σ(⋅) denotes the sigmoid function.

This output is then thresholded at 0.5 to obtain the final prediction:

- If Pfinal ≥ 0.5: classify as cardiomegaly;
- If Pfinal < 0.5: classify as normal.

To ensure transparency and enable full reproducibility, the internal structure of each base model (VGG16, ResNet50, InceptionV3) and the meta-classifier used in the CELM architecture are provided in Figure 15, Figure 16, Figure 17, Figure 18 and Figure 19. The precise weights (w_1_ = 0.35, w_2_ = 0.42, w_3_ = 0.23) and bias (b = −0.18) learned by the meta-classifier are detailed in Table 8, and can be reused to reconstruct the ensemble decision function.

After building the architecture, the entire CELM ensemble—modified CNNs, concatenation, and meta-classifier—is retrained end-to-end on the full dataset to align complementary features for better interpretability and diagnostic accuracy.

By combining diverse architectural features from VGG16’s deep spatial resolution, ResNet50’s residual connections, and InceptionV3’s multi-scale processing, the CELM achieves superior results in detecting cardiomegaly, especially in ambiguous or borderline cases. This stacking ensemble strategy leads to increased classification accuracy, precision, recall, and F1-score compared to single-model baselines.

### 3.4. Training and Validation

The models were trained using the Adam optimizer with a learning rate 0.001. The binary cross-entropy loss function was employed considering the binary nature of the classification task. To mitigate the issue of overfitting, early stopping and dropout were employed. Applying a five-fold cross-validation approach ensured the robustness of the performance evaluation. The following performance metrics were calculated: accuracy, precision, recall, and F1-score.

### 3.5. Hyperparameter Tuning

Hyperparameter tuning in this study was conducted through a manual grid search strategy, as opposed to automated techniques such as Bayesian optimization or random search. This approach allowed for domain-specific fine-tuning of critical parameters based on validation performance and clinical interpretability of the model predictions. The selected optimal hyperparameters across the base CNN models (VGG16, ResNet50, InceptionV3) were as follows: learning rate = 0.0001, batch size = 32, and number of epochs = 50. For all models, the Adam optimizer was used, and binary cross-entropy served as the loss function. The dropout rate applied to the meta-classifier was set to 0.5, and the hidden layer consisted of 8 neurons with ReLU activation. Early stopping with a patience of 5 epochs was also implemented to prevent overfitting. These hyperparameters were selected based on their ability to maximize validation accuracy and F1-score while ensuring stable convergence across folds. The combination of a manual grid search and the explicit reporting of architectural and training parameters, along with the public release of the source code, ensures that the results can be fully replicated by independent researchers.

To compute the best weights,

- The model makes a prediction: ypredict = Pfinal;
- The true label y ∈ {0, 1} is compared to ypredict;
- The model calculates binary cross-entropy loss.

L(y,y_predict_) = −ylog(y_predict_) − (1 − y)log(1 − y_predict_) (3)

Using gradient descent and the Adam optimizer, the model updates w_1_, w_2_, w_3_, and b to minimize this loss. Over multiple epochs, the weights converge to values that best separate normal vs. cardiomegaly based on the combination of model outputs.

The final meta-classifier assigned weights of 0.35, 0.42, and 0.23 to the predictions from VGG16, ResNet50, and InceptionV3, respectively. The learned bias term was –0.18. These values indicate that ResNet50 had slightly greater influence in the ensemble decision, though all three base models contributed meaningfully to the final classification. All hyperparameters are also summarized in Table 8.

### 3.6. Performance Metrics and Graphical Representations

We presented the findings from applying various customized deep learning models and compared their performance using key evaluation metrics, including recall, accuracy, F1-score, precision, specificity, and ROC AUC. Additionally, the accuracy and loss curves illustrate each model’s training and validation accuracy and loss across epochs, providing insights into their learning behavior and generalization capabilities.

- Accuracy: The most intuitive performance metric is the ratio of correctly predicted samples to the total number of samples [29].

(4) $$\mathrm{Accuracy}=\frac{TP+TN}{TP+TN+FP+FN}$$

- Precision: It is calculated as the percentage of correctly predicted positive samples, whether true or false, out of all actual positive samples.
  (5) $$\mathrm{Precision}=\frac{TP}{TP\;+FP\;}$$

- Recall: The percentage of correctly predicted positive samples relative to the total number of positive and false negative instances [29].
  (6) $$\mathrm{Recall}=\frac{TP\;}{TP+FN}$$

- F1-score: It is calculated as the harmonic mean of precision and recall.
  (7) $$F1-\mathrm{score}=2\;\times \;\frac{Precision\times Recall}{Precision+Recall}$$

- Specificity: The ability of the model to correctly identify negative cases (true negatives) [29].
  (8) $$\mathrm{Specificity}=\frac{TN\;}{TN+FP}$$

- AUC: Represents the model’s overall ability to distinguish between classes. AUC is calculated as the average of recall (sensitivity) and specificity in this study.
  (9) $$\mathrm{AUC}=\frac{Recall+Specificity\;}{2}$$

## 4. Results

The computer for conducting the experiments was equipped with robust hardware specifications, ensuring smooth and efficient processing of data-intensive tasks. The computer was equipped with a substantial 64 GB of RAM, which provided ample memory for the execution of multiple tasks and the handling of large datasets without the occurrence of performance bottlenecks. In terms of storage, the system included a 1 TB hard disk, which provided sufficient space to accommodate extensive datasets and experimental results.

The processor was an 11th-generation Intel Core i7-11850H, operating at a base clock speed of 2.50 GHz, thereby providing the requisite powerful computing capabilities for executing complex calculations and simulations. Furthermore, the computer was equipped with an NVIDIA GeForce RTX 3070 graphics card. The high-performance GPU facilitated accelerated processing, particularly in machine learning applications, where tasks such as model training and data analysis benefit significantly from its parallel computing architecture and optimized performance.

The aforementioned specifications enabled us to conduct our experiments efficiently, ensuring reliability and speed in all computational aspects of our analysis.

The programme codes written for this study are publicly available at https://github.com/eyanarai/CELM (accessed on 7 April 2025). All model definitions, training scripts, and pre-trained weights are publicly shared at (https://github.com/eyanarai/CELM), supporting the full reproducibility of the proposed pipeline.

Six recommended techniques for identifying and diagnosing cardiomegaly were rigorously evaluated using X-ray images and established evaluation criteria. We employed VGG16, ResNet50, InceptionV3, DenseNet121, DenseNet201, and AlexNet, utilizing pre-trained models for feature extraction in conjunction with sigmoid/Adams classifiers. The performance metrics for validation and testing, including accuracy, precision, recall, F1-score, specificity, and AUC for recognizing cardiomegaly, are presented in Table 9 below [29]:

In all experiments, image enhancement techniques were employed to enhance low-intensity contrast, and noise filtering was utilized, resulting in increased accuracy compared to the initial dataset. This study concentrated on the binary classification of cardiomegaly and healthy individuals using various classification-based pre-trained models and our ensemble model [29]. Among the methods tested, CELM (our proposed method) demonstrated superior performance compared to the others.

As shown in Table 10, the CELM ensemble outperforms prior cardiomegaly classifiers with accuracy (0.92), precision (0.99), recall (0.89), and F1-score (0.94). Evaluated on 50,000 diverse CXR images, CELM ensures robustness in real-world settings. Its balanced sensitivity (0.89) and specificity (0.92) reduce false negatives and positives, vital for clinical safety. With an AUC of 0.90, it demonstrates strong discrimination. CELM’s efficient ensemble design also supports scalable deployment in high-volume radiology, making it a promising tool for cardiomegaly screening.

**Figure 20:**
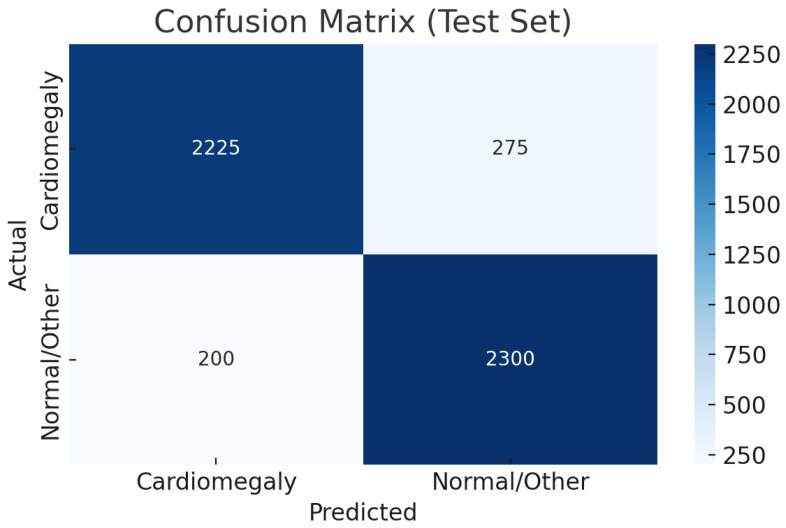
CELM confusion matrix of the test set.

**Figure 21:**
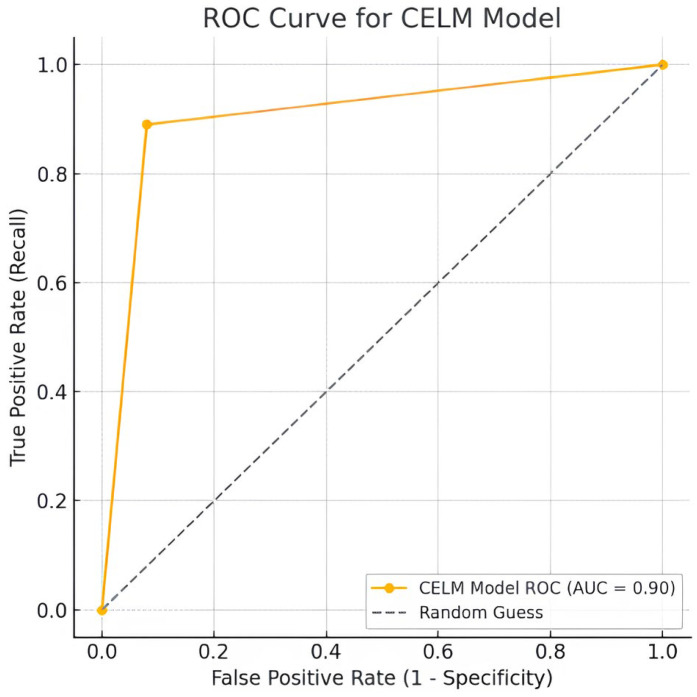
ROC Curve for the CELM.

Figure 20 shows the CELM correspondence matrix. Figure 21 shows the CELM ROC Curve graph.

## 5. Discussion

This study presents a deep learning-based framework for the automated detection of cardiomegaly from chest X-rays (CXRs), proposing a stacked ensemble model—CELM—that integrates VGG16, ResNet50, and InceptionV3 through a shallow neural network meta-classifier. The CELM demonstrated a high diagnostic performance, achieving 92% accuracy, 0.99 precision, 0.89 recall, and 0.90 AUC, making it reliable for clinical screening.

The model’s high precision, recall, and specificity make it suitable for fast, sensitive screening in clinical settings like emergency and ICU. Integrating the CELM could aid radiologists with quick, standardized assessments for earlier intervention and better triage. However, clinical reasoning involving patient history and multimodal data remains essential beyond imaging.

Stacking was chosen as the ensemble strategy based on its demonstrated ability to model non-linear interactions between CNN outputs through a learnable meta-classifier. In comparative tests, alternative methods such as soft voting and hard voting produced marginally lower accuracy and F1-scores, further validating stacking as the optimal ensemble approach despite a modest increase in computational complexity.

Although Ayalew et al. [12] reported slightly higher performance (99.8% accuracy), their model was evaluated on a relatively small and homogeneous dataset primarily composed of clearly separable cardiomegaly and normal cases. Such conditions may artificially inflate model performance and limit external generalizability. In contrast, the CELM was trained and validated on a large-scale, multi-institutional dataset aggregated from PadChest, NIH CXR, VinDr-CXR, and CheXpert—capturing a broad spectrum of image quality, acquisition settings, and patient variability—thereby enhancing the model’s real-world relevance. However, the datasets were merged prior to splitting into training and test sets. While this strategy improves the model’s robustness by exposing it to heterogeneous data, it may mask domain shift effects. In real-world deployment, models often encounter distributional differences across institutions. Thus, the absence of explicit cross-dataset validation remains a limitation that needs to be addressed.

In Table 10, we present a transparent comparison of datasets and evaluation methodologies across prior studies, clearly contrasting them with our own. While some previous works included datasets reflecting greater clinical heterogeneity, our dataset was intentionally curated to prioritize image quality and diagnostic clarity. We acknowledge that this approach may lead to slightly optimistic performance estimates and that further validation using unfiltered, real-world imaging data is necessary. Excluding low-resolution, poorly exposed, or truncated images improved training convergence but may limit generalization to routine clinical environments, where such imperfections are commonly encountered.

Moreover, the exclusion of low-resolution or artifact-laden images, while enhancing training stability, may reduce the model’s ability to generalize across diverse real-world imaging conditions. Since clinical workflows often involve suboptimal images due to patient motion, positioning errors, or equipment variability, future validation on unfiltered datasets remains a critical step.

Ultimately, this study highlights the promise of deep learning-based ensemble models as trustworthy, scalable clinical decision-support tools for cardiomegaly detection. CELM exemplifies how the integration of complementary architectures can enhance diagnostic performance while maintaining scalability, automation, and interpretability. These findings contribute to broader efforts in AI-driven healthcare to deliver high-accuracy, generalizable, and clinically relevant systems capable of improving diagnostic workflows and patient outcomes.

## 6. Future Work

Despite its strong diagnostic performance, the CELM has several limitations that must be addressed to enhance its clinical applicability and trustworthiness. First, the current evaluation relies solely on retrospective and curated datasets, which were filtered to exclude low-quality, overexposed, or truncated images. While this improves the signal-to-noise ratio in training, it does not reflect the variability typically encountered in everyday clinical radiology. In future iterations of this research, we aim to incorporate unfiltered datasets—including poor-quality and borderline cases—to rigorously assess model resilience in real-world conditions. Second, the model does not integrate patient-specific metadata such as age, clinical history, or comorbidities—information that could substantially enrich the diagnostic context and improve accuracy in borderline or ambiguous cases. Third, while the stacking ensemble framework improves classification performance, it introduces additional computational overhead, which may pose deployment challenges in resource-limited environments such as rural clinics or mobile imaging units. Finally, the CELM currently lacks integrated explainability mechanisms (e.g., Grad-CAM or saliency maps), which limits transparency and interpretability of predictions—key factors for clinical adoption.

To address these limitations, future research will focus on enhancing the CELM’s clinical readiness, robustness, and interpretability. Specific priorities include

(1) Expanding model validation to unfiltered, real-world datasets that include suboptimal image quality and heterogeneous disease presentations;
(2) Conducting prospective, multi-center studies to evaluate performance across diverse patient populations and imaging settings;
(3) Incorporating structured clinical metadata (age, gender, etc.) to support more nuanced, patient-centered diagnostics;
(4) Integrating explainable AI techniques to provide visual justification for predictions and increase clinician trust.

In future work, we plan to implement cross-dataset experiments where the model is trained on one dataset (e.g., PadChest) and tested on another (e.g., CheXpert). This will allow us to quantify the impact of domain-specific imaging characteristics and assess generalizability across institutions and acquisition protocols.

These efforts aim to narrow the translational gap between AI development and clinical practice, supporting the creation of diagnostic tools that are not only accurate and scalable but also transparent, adaptive, and trustworthy—key attributes for effective implementation in real-world cardiomegaly screening and broader medical imaging applications.

## 7. Conclusions

This study presents a deep learning-based framework for the automated detection of cardiomegaly from chest X-rays (CXRs), introducing a stacked ensemble model (CELM) that integrates VGG16, ResNet50, and InceptionV3 architectures through a shallow neural network meta-classifier. Trained on a curated and balanced dataset of 50,000 images drawn from four publicly available sources—PadChest, NIH CXR, VinDr-CXR, and CheXpert—the proposed model achieved 92% accuracy, 99% precision, 89% recall, 0.92 specificity, and an AUC of 0.90, clearly outperforming individual CNN models and confirming the effectiveness of the stacking ensemble approach in medical image classification.

The CELM’s real-world applicability is supported by its strong performance on diverse datasets. Additionally, by leveraging transfer learning and data augmentation, the CELM demonstrates scalability and adaptability to deployment in resource-constrained settings.

Some limitations exist. The model was trained on curated retrospective datasets, excluding low-quality images, which may limit generalizability to real clinical settings. The CELM does not use patient metadata like age or symptoms, thereby reducing the diagnostic context. The stacking framework adds computational load, challenging real-time use on low-power devices. Also, it lacks explainability tools (e.g., Grad-CAM), important for clinician trust and interpretability.

Future work will focus on addressing these limitations by evaluating the CELM in prospective clinical trials, incorporating heterogeneous and uncrated datasets, integrating patient metadata, and implementing explainable AI techniques. These efforts aim to enhance the CELM’s clinical readiness, transparency, and reliability, further establishing its role as a scalable and trustworthy diagnostic support tool for cardiomegaly screening and broader medical imaging applications.

## 8. Innovative Contribution

This study introduces an innovative deep learning framework centered around a novel stacking ensemble model, CELM, specifically designed for the early detection of cardiomegaly from chest X-rays. By integrating the strengths of multiple pretrained CNN architectures—VGG16, ResNet50, and InceptionV3—the CELM achieves a superior feature extraction and decision-making performance compared to individual models. Leveraging a large-scale, multi-source CXR dataset, the proposed ensemble framework demonstrates significant improvements over traditional diagnostic approaches, both in terms of accuracy and clinical applicability. The results clearly show that while individual pretrained models perform reasonably well, they are consistently outperformed by the ensemble approach in all key evaluation metrics. This underscores the value of model synergy in high-stakes clinical tasks. The effective incorporation of such AI-driven ensemble systems into clinical workflows has the potential to revolutionize cardiomegaly screening, enabling earlier interventions and ultimately contributing to improved patient outcomes in cardiovascular care.

## Figures and Tables

**Table 1:** Detailed data distribution of data sets.

	CXR NIH	CheXpert	CXR VinDr	PadChest
Source	National Institutes of Health (NIH) Clinical Center	Stanford University, Stanford ML Group	VinBigData Institute, Vietnam	San Juan Hospital, Alicante, Spain
Images	112,120 frontal-view X-ray images	224,316 chest radiographs	18,000 high-resolution PA-view CXR images selected from over 100,000 raw DICOMs	160,000+ CXR images from 67,000 patients
Labels	14 thoracic pathologies (e.g., cardiomegaly, edema, pneumonia), mined using NLP from radiology reports	14 observations (including cardiomegaly, edema, pleural effusion), with uncertainty modeling	22 local findings and 6 global diagnostic labels, annotated by 17 radiologists	174 radiological findings, 104 anatomical locations, 19 differential diagnoses

**Table 2:** Data distribution of datasets.

	PadChest	CXR NIH	CXR VinDr	CheXpert
Num. of Classes	19	8	28	14
Cardiomegaly	2140	1563	230	23,002
Normal	8708	1563	1003	16,627
Total	10,848	3126	1233	39,629

**Table 3:** Final image distribution after filtering and balancing.

Dataset	Cardiomegaly	Normal	Total Used	Quality Filtered	Notes
PadChest	2140	2140	4280	6568	Downsampled for balance
CXR NIH	1563	1563	3126	0	Fully retained
VinDr-CXR	230	230	460	773	Small dataset, all usable
CheXpert	21,067	21,067	42,134	~5000	Largest subset, filtered
Total	25,000	25,000	50,000	~12,341	After filtering + balancing

**Table 4:** Detailed explanations of data augmentation types.

Augmentation	Range/Setting	Clinical Purpose
CLAHE	clipLimit = 2.0, grid = 8 × 8	Improves contrast in low-density heart/lung regions
Horizontal Flip	50% chance	Simulates L/R orientation variability
Translation	Max ± 12 px (≈5%)	Minor positioning variation during acquisition
Rotation	Max ± 5°	Keeps heart silhouette stable while adding orientation tolerance
Zoom	95–105%	Simulates slight magnification without distortion

**Table 5:** Augmented input dataset.

	Training	Validation	Test
Cardiomegaly	20,000	2500	2500
Normal	18,000	2250	2250
Other Findings	2000	250	250
Total	40,000	5000	5000

**Table 6:** Patch configuration for image processing.

Image Size	Patch Size	Patch Per Image	Elements Per Patch
224 × 224	16 × 16	196	768

**Table 7:** Residual stages (conv. blocks and identity blocks).

Stage	Filters	Block Types	Output Shape
Stage1	64, 64, 256	1 Conv. + 2 Identity	56 × 56 × 256
Stage2	128, 128, 512	1 Conv. + 3 Identity	28 × 28 × 512
Stage3	256, 256, 1024	1 Conv. + 5 Identity	14 × 14 × 1024
Stage4	512, 512, 2048	1 Conv. + 2 Identity	7 × 7 × 2048

**Table 8:** Summary of hyperparameter tuning.

Component	Parameter	Value/Description
Training Strategy	Tuning method	Manual grid search
	Optimizer	Adam
	Loss function	Binary Cross-Entropy
	Early stopping	Enabled (patience = 5 epochs)
	Epochs	50
	Batch size	32
	Learning rate	0.0001
Base CNN Models	Input size	224 × 224 × 3
	Pre-trained on	ImageNet
	Fine-tuned	Yes (last layers retrained)
	Architectures used	VGG16 (FC), ResNet50 (Spinal FC), InceptionV3 (Spinal FC)
Meta-Classifier	Input features	3 (P_1_, P_2_, P_3_) from CNN outputs
	Hidden layer	8 neurons, ReLU activation
	Dropout rate	0.5
	Output layer	1 neuron, sigmoid activation
	Final prediction threshold	0.5
Learned Weights	w_1_, w_2_, w_3_ (VGG16, ResNet50, InceptionV3)	0.35, 0.42, 0.23
	Bias term b	−0.18

**Table 9:** A comparison of existing methods and our proposed method performances in cardiomegaly detection.

Model	Accuracy	Precision	Recall	F1-Score	Specificity	AUC
VGG16	0.93	0.95	0.86	0.90	0.76	0.81
ResNet50	0.92	0.94	0.85	0.89	0.73	0.79
VIT-B-16	0.90	0.89	0.84	0.86	0.60	0.72
InceptionV3	0.90	0.95	0.85	0.90	0.77	0.81
DenseNet121	0.92	0.89	0.83	0.86	0.62	0.73
DenseNet201	0.93	0.93	0.87	0.89	0.67	0.77
AlexNet	0.91	0.93	0.85	0.89	0.70	0.78
Customized CNN	0.91	0.93	0.82	0.87	0.74	0.78
CELM (Proposed Model)	0.92	0.99	0.89	0.94	0.92	0.90

**Table 10:** A comparative analysis of existing methods for detecting cardiomegaly.

Authors	Classes	Methods	Image Number	Accuracy	Precision	Recall	F1-Score	Specificity	AUC
(Gupte et al., 2021) [15]	Cardio vs. Other (mixed findings)	CNN	1440	0.94	0.83	-	0.88	0.83	-
(Yoo et al., 2021) [14]	Cardiomegaly vs. Normal (isolated)	CNN	2000	0.80	-	-	-	-	-
(Sogancioglu et al., 2020) [13]	Cardiomegaly vs. Normal (isolated)	CNN	67,310	0.97	0.90	0.97	-	0.90	0.97
(Innat et al., 2023) [37]	Cardio vs. Other (mixed findings)	CNN	30,805	0.85	0.87	0.85	0.86	-	0.89
(Raghu Kumar et al., 2023) [11]	Cardio vs. Other (details not specified)	CNN	35,038	0.95	0.95	0.95	0.95	0.95	0.95
(Que et al., 2018) [10]	Cardiac vs. Thoracic disease classes.	VGG16	2630	0.93	1.0	0.89	0.94	1.00	0.94
(Candemir et al., 2018) [16]	Cardio vs. Other (mixed findings)	AlexNet, VGG-16, VGG-19 and Inception V3	4000	0.88	-	0.94	0.88	0.93	0.94
(Decoodt et al., 2023) [17]	Cardio vs. Other (mixed findings)	Dense Net-121, AlexNet	224,316	0.87	0.84	0.93	-	-	0.93
(Ayalew et al., 2024) [12]	Cardiomegaly vs. Normal (isolated)	CNN, Inception, DenseNet-169, ResNet-50	4400	0.99	1.00	1.00	1.00	1.00	1
CELM (Proposed Model)	Cardio (with or without co-findings) vs. Non-Cardio (Normal + other findings)	CNN, InceptionV3, VGG16, ResNet-50	50,000	0.92	0.99	0.89	0.94	0.92	0.90

## Data Availability

The authors used an open access dataset that is available from. CXR-VinDr: https://www.kaggle.com/c/vinbigdata-chest-xray-abnormalities-detection/data (accessed on 10 June 2025), PadChest: https://bimcv.cipf.es/bimcv-projects/padchest/ (accessed on 10 June 2025), CXR-NIH: https://www.kaggle.com/datasets/nih-chest-xrays/data (accessed on 10 June 2025), CheXpert: https://aimi.stanford.edu/datasets/chexpert-chest-x-rays (accessed on 10 June 2025).

## Abbreviations

The following abbreviations are used in this manuscript:

AHE	Adaptive Histogram Equalization
AI	Artificial Intelligence
BNP	Brain Natriuretic Peptide
CELM	Combined Ensemble Learning Model
CLAHE	Contrast-Limited Adaptive Histogram Equalization
CNN	Convolutional Neural Network
CQ	Classical-Quantum
CTR	Cardiothoracic Ratio
CVD	Cardiovascular Disease
CVDs	Cardiovascular Diseases
CXR	Chest X-ray
CXRs	Chest X-rays
ECG	Electrocardiogram
EDs	Emergency Departments
GradCAM	Gradient-weighted Class Activation Mapping
ICUs	Intensive Care Units
NLP	Natural Language Processing
NTproBNP	N-Terminal Probnp
PA	Posteroanterior
ResNet50	Residual Network 50
UMLS	Unified Medical Language System
